# Tetraspanin 1 (TSPAN1) promotes growth and transferation of breast cancer cells via mediating PI3K/Akt pathway

**DOI:** 10.1080/21655979.2021.2003130

**Published:** 2021-12-02

**Authors:** Yange Wu, Wenxiu Chen, Yufeng Gong, Hongxia Liu, Bo Zhang

**Affiliations:** Department of Pathology, Pingshan District People’s Hospital of Shenzhen, Pingshan General Hospital of Southern Medical University, Shenzhen, Guangdong Province, China

**Keywords:** Breast cancer, tetraspanin 1 (tspan1), growth, epithelial-mesenchymal transition (EMT), PI3K/Akt pathway

## Abstract

The incidence and mortality of breast cancer rank first among all types of female tumors. To improve patients’ prognosis with advanced breast cancer, new and more effective targets still need to be explored and identified. Tetraspanin 1 (TSPAN1) is highly expressed in several cancers and affects the progression of these tumors. However, there are few studies focused on its role in breast cancer. Previous study showed that TSPAN1 promoted epithelial-mesenchymal transition (EMT) and metastasis, and whether TSPAN1 could promote breast cancer via regulating EMT needs further study. In this study, we found high TSPAN1 expression in breast cancer tumor samples and cell lines which was confirmed by bioinformation analysis. The ablation of TSPAN1 suppressed the growth, and motility of breast cancer cells. We further found that TSPAN1 affected the EMT and mediated the PI3K/Akt pathway in breast cancer cells. In addition, TSPAN1 depletion suppressed tumor growth of breast cancer in mice. In summary, we thought TSPAN1 suppressed growth and motility of breast cancer via mediating EMT and PI3K/AKT pathway, and could serve as a potential target for treatment of breast cancer.

## Introduction

As a common malignant tumor in women, the incidence and mortality of breast cancer rank first among all types of female tumors, posing a serious threat to women’s health [1,2]. Compared with western developed countries, the incidence of breast cancer in China has been increasing [3,4]. In particular, breast cancer has become the most common malignant tumor among women in large cities [5]. When various external stimuli (various carcinogenic factors) act on mammary epithelial cells, the cells will produce a variety of stress responses, resulting in cell growth, which leads to breast cancer [6]. At present, the clinical treatment of breast cancer is mainly surgical surgery, but the clinical surgical resection range is wide, the trauma is large, and many postoperative complications often occur, leading to serious psychological harm to patients [7,8]. In recent years, more and more attention has been paid to targeted treatment for breast cancer [9]. In order to improve the prognosis of patients with advanced breast cancer, new and more effective targets still need to be explored.

Tetraspanin 1 (TSPAN1) is a member of the TSPAN superfamily and consists of four transmembrane domains that interact with a variety of cell surface signaling molecules [10,11]. The TSPAN superfamily has been reported to influence the malignant properties of cancer cells, including growth, apoptosis, metastasis, and invasion [12,13]. TSPAN1 is highly expressed in variety of cancers [13–15]. However, there are few studies on the role of TSPAN1 in breast cancer.

Epithelial-mesenchymal transition (EMT) is a complex and transient process that causes tumor epithelial cells to lose their polarity, and increases the cells’ ability to invade and migrate [16]. The changes of adhesion molecules and ECM are two key factors in EMT process [17]. Previous study showed that TSPAN1 promoted EMT and metastasis of cholangiocarcinoma via promoting PI3K/AKT pathway, suggesting that TSPAN1 may be a potential therapeutic target for cholangiocarcinoma [18].

In this study, we speculated that TSPAN1 could promote breast cancer by promoting the occurrence of EMT. This study aims to uncover the role of TSPAN1 in the progression of breast cancer and uncover the possible mechanism.

## Materials and methods

### Bioinformation analysis

Biological information was obtained to investigate the mRNA levels of TSPAN1 in breast cancer and normal tissues from TCGA database (https://www.cancer.gov/about-nci/organization/ccg/research/structural-genomics/tcga).

### Antibodies and siRNAs

TSPAN1 (1:500 dilution for Immunoblot, 1:100 for IHC, PA5-21,973, Thermo Fisher), Ki67 (1:200 dilution for IHC, ab15580, Abcam), E-cadherin (1:1000 dilution, ab76055, Abcam), N-cadherin (1:500 dilution, ab76011, Abcam), Vimentin (1:200 dilution, ab92547, Abcam), AKT (1:1000 dilution, ab8805, Abcam), p-AKT (phospho T308, 1:500 dilution for Immunoblot, 1:50 for IHC, ab38449, Abcam), PI3K (1:1000 dilution, ab140307, Abcam), p-PI3K (phospho Y458, 1:500 dilution, ab278691, Abcam), and GAPDH (1:2000 dilution, ab8245, Abcam) antibodies were obtained and diluted as indicated.

Two TSPAN1 siRNAs (#1 and #2) and control siRNA were bought from Riobio (Guangzhou, China) and used with the final concentration of 20 μM.

### Patients and samples

Patients diagnosed clinically and pathologically standards with breast cancer in Pingshan District People’s Hospital of Shenzhen were included in this study. This study was approved by the Medical Ethics Committee of Pingshan District People’s Hospital of Shenzhen (Approval no.2019144). Tumor and adjacent non-tumor tissue specimens were used for the IHC and Immunoblot assays.

### Immunohistochemistry staining

Tumor and adjacent non-tumor tissue specimens were fixed with 10% formalin solution, embedded in paraffin, dewaxed with xylene at 65°C, and then rehydrated in a gradient ethanol series. The samples were immersed in citrate buffer (pH, 6.0) at 98°C for 30 min and placed in a microwave for incubation for 10 min for antigen retrieval. Then, hydrogen peroxide was added to block endogenous peroxidase activity and the samples were incubated at room temperature for 10 min, followed by blocking with 2% BSA (Sigma-Aldrich; Merck KGaA) for 20 min at room temperature. Then sections were incubated with antibodies at 4°C for 2 h, followed by the secondary antibody incubation (rabbit; 1:200 dilution; cat. no. ab205718; Abcam) at 37°C for another 2 h. Sections were stained with 3,3-diaminobenzidin (DAB) for 10 min and images were observed by a microscopy (Olympus).

### Cell culture and transfection

Human breast cancer cell lines including MCF-7 (HTB-22), T47-D (HTB-133), MDA-MB-231 (HTB-26), and SK-BR-3 (HTB-30), and normal breast cell line MCF-10A (CRL-10317) were purchased from ATCC. These cells were all cultured in RPMI-1640 medium containing 1% penicillin-streptomycin (P/S) with 10% FBS. All cell lines were grown at 37°C with 5% CO_2_. A total of 1 × 10^5^ cells per well were seeded into 6-well plates and a total of 2 groups were used: siRNA group (5 μL) and si-NC group (5 μL). Knockdown efficiency was measured by both reverse transcription-quantitative PCR (RT-qPCR) and western blot analysis after 48 h. The TSPAN1 siRNA group sequences were:

5ʹ‑GGCUCACGACCAAAAAGUAtt‑3ʹ (sense) and 5ʹ‑UACUUUUUGGUCGUGAGCCtt‑3ʹ (antisense). The scramble siRNA group sequences were: 5ʹ‑UUCUCCGAACGUGUCACGUdTdT‑3ʹ (sense), 5ʹ‑ACGUGACACGUUCGGAGAAdTdT‑3ʹ (antisense).

### RT-qPCR

The total RNA was extracted using TRIzol® (Invitrogen; Thermo Fisher Scientific, Inc.) and 2 µg was reversely transcribed to cDNA template by the M-MuLV cDNA Synthesis Kit (Sangon, China). RT-qPCR was performed on a Smart Cycler using FastSYBR Mixture (Sangon, China). Primer sequences are listed as follow: TSPAN1 (Forward) 5ʹ-CCAATAAGCTTATGCAGCTTCAATTAAGA −3ʹ, and (Reverse) 5ʹ – CCAATGAATTCTTGTAGATTGCAGTACAGATACATG-3ʹ; GAPDH (Forward) 5ʹ-TGATGACATCAAGAAGGTGGTGAAG −3ʹ, and (Reverse) 5ʹ-TCCTTGGAGGCCATGTGGGCCAT −3ʹ. The primers were checked on primer 5.0 software. The following thermocycling conditions were used: Initial denaturation at 95°C for 3 min; followed by 30 cycles of denaturation at 95°C for 30 sec, annealing at 58°C for 30 sec and extension at 72°C for 30 sec. The 2-ΔΔCq method was used to quantify the results.

### Immunoblotting

Proteins were extracted from cells or tissues with lysis buffer [60 mM Tris-HCl (pH 6.8), 2% SDS, 20% glycerol, 0.25% bromophenol blue, 1.25% 2-mercaptoethanol and protease inhibitor cocktail]. Protein concentration was determined using the BCA method. Lysates were then isolated by 8% SDS-PAGE (15 ug/mL proteins were loaded in each line) and transferred onto Nitrocellulose (NC) membrane (Thermo, American). Membranes were blocked by 5% fat-free milk and incubated with the primary antibodies at 4°C for another 2 h. The NC membranes were incubated with the horseradish peroxidase (HRP)-conjugated secondary antibodies (rabbit; 1:5,000 dilution; cat. no. ab205718; mice; 1:5,000 dilution; cat. no. ab6789; Abcam) for 1 h and visualized by chemiluminescence.

### CCK-8 assay

CCK-8 (ab228554) assays were performed to detect the capacity of cell proliferation. 1000/well of MCF-7 or SK-BR-3 cells were plated in 96-well plates and cultured at 37°C. Then, 10 μl of cell counting kit-8 (CCK-8) solution was added in each well for 3 h. Subsequently, the absorbance was measured using a microplate reader (UV-3600 Shimadzu analysis) at 450 nm wavelength.

### Colony formation assay

Both MCF-7 or SK-BR-3 cells plated in 6-well plates (1000 cell/well) were transfected with siRNAs for 48 h and maintained for 14 days. Then the cells were stained with 0.2% crystal violet for 20 min and photographed.

### Wound healing assay

Wound healing assay was performed to detect the capacity of cell migration. 10^5^/well of MCF-7 or SK-BR-3 cells were plated in 24-well plates. When reached 100% confluency, a pipette tip was used to product the scratch. After 24 h incubation with serum-free medium, the width between the edges of the scratch was calculated. Migration ability was measured using ImageJ software (v1.8.0; National Institutes of Health) and quantified as a percentage of wound width (post-healing wound width/pre-healing wound width).

### Transwell assay

Transwell assay was conducted to investigate the invasion of cells. 10^6^ cells were seeded into the upper chambers of transwell chamber (8 um, BD, coated with 20% matrigel) with 100 µl medium without serum and complete medium was added into the low chambers. The cells were cultured at 37°C with 5% CO_2_ for 24 h and fixed by 4% paraformaldehyde (PFA) for 30 min and stained with 0.2% crystal violet for 10 min. Then, images were captured, and the number of invaded cells were counted using an Olympus inverted fluorescence microscope (IX71; Zeiss AG).

### Xenograft study

The animal experiment protocol was approved by the Medical Ethics Committee of Pingshan District People’s Hospital of Shenzhen (Approval no.2019144). 12 female 6-week-old BALB/c nude mice were purchased from Beijing Vital River. MCF-7 cells transfected with control or TSPAN1 siRNAs were inoculated into the upper back of mice. Tumor growth was monitored every 7 days and the tumor volumes were calculated. After 35 days, tumors were harvested, and mice were sacrificed. The tumor tissues were used for subsequent IHC experiments.

### Statistical analysis

All the data were analyzed by SPSS version 22.0. The quantitative data were assessed as mean ± SEM, and student’s t-test was used to analyze the two groups. Differences in TSPAN1 expression in cell lines were analyzed using one-way ANOVA. *P* < 0.05 was considered to be statistically significant.

## Results

### TSPAN1 was high expression in human breast cancer

**Figure 1.**
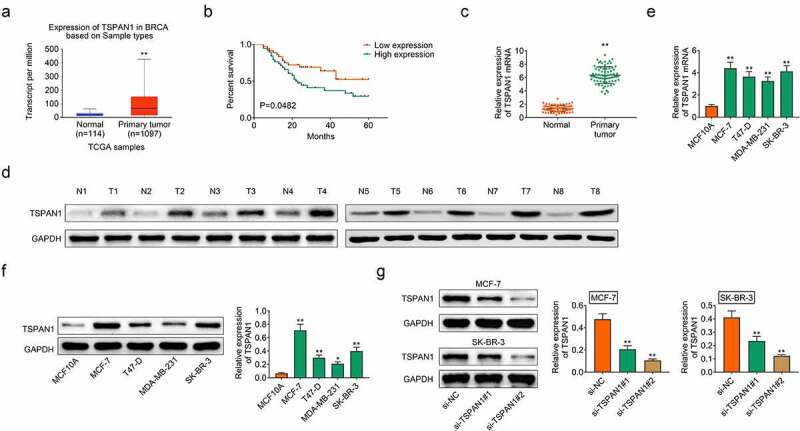
**TSPAN1 was high expression in human breast cancer**. (a). TCGA database showed the transcript per million of TSPAN1 in breast cancer tissues and normal tissues. (b). The survival analysis of patients with high or low TSPAN1 expression in tumor tissues. (c). qPCR assays showed the mRNA levels of TSPAN1 in breast cancer tissues and adjacent tissues. (d). Immunoblot assays showed the protein levels of TSPAN1 in 8 pairs of breast cancer tissues and adjacent tissues. (e). Immunoblot assays showed the protein levels of TSPAN1 in normal breast cell line MCF-10A, and four types of breast cancer cell lines, including MCF-7, T47-D, MDA-MB-231, and SK-BR-3. (f). Immunoblot showed the expression of TSPAN1 in MCF-7 and SK-BR-3 cells upon the transfection of control or TSPAN1 siRNAs. Data are shown as mean ± SEM, *p < 0.05, **p < 0.01

To clarify the possible effects of TSPAN1 on breast cancer progression, we first evaluated its expression levels via bioinformation analysis. Through the TCGA database, we noticed the transcript per million of TSPAN1 in breast cancer tissues was obviously higher (Figure 1a). We further noticed the expression of TSPAN1 in tumor tissues was correlated with the survival rates of patients (Figure 1b). We also found the high mRNA levels in breast cancer tissues (Figure 1c). Similarly, immunoblot assays confirmed the high TSPAN1 protein levels in 8 tumor tissues of breast cancer patients (Figure 1d).

Then the patients were divided into TSPAN1 high and low expression groups according to the staining levels of TSPAN1 in tumor tissues. Through the clinical-pathological analysis, we noticed the expression of TSPAN1 was significantly correlated with pathological staging and lymph node metastasis of patients (Table 1). However, no obvious correlations were found between TSPAN1 expression and the clinical features including age, tumor size, et al (Table 1).

**Table 1.** The relationship between the expression level of TSPAN1 and the clinicopathological characteristics in breast cancer

Characteristics	Number of patients	TSPAN1	TSPAN1	P value
		High expression (< median)	Low expression (≥ median)	
Number	75	37	38	
Ages(years)				0.563
≤60	38	20	18	
>60	37	17	20	
Tumor size(cm)				0.396
≤2	30	13	17	
>2	45	24	21	
HER-2 status				0.404
Negative	43	23	20	
Positive	32	14	18	
ER status				0.127
Negative	33	13	20	
Positive	42	24	18	
PR status				0.42
Negative	37	20	17	
Positive	38	17	21	
Pathological staging				0.036*
&Iota;	14	4	10	
&Iota;&Iota;	25	9	16	
&Iota;&Iota;&Iota;	20	13	7	
IV	16	11	5	
Lymph node metastasis				0.044*
No	31	11	20	
Yes	44	26	18	

*p < 0.05

We then further detected the expression of TSPAN1 in breast cancer cell lines. Four types of breast cancer cell lines, including MCF-7, T47-D, MDA-MB-231, and SK-BR-3 were used in this experiment. Consistently, immunoblot results showed the high TSPAN1 expression in breast cancer cell lines (Figure 1e). Therefore, these data indicated that TSPAN1 was upregulated in human breast cancer.

## TSPAN1 contributes to the growth and motility of breast cancer cells

Then we performed the in vitro function experiments to investigate the effects of TSPAN1 on the growth and motility of breast cancer cells. We used two siRNAs of TSPAN1 to knockdown its expression in two types of breast cancer cells, MCF-7 and SK-BR-3 cells. Immunoblot assays results showed the obviously decreased expression of TSPAN1 in TSPAN1-siRNA transfected cells, suggesting the effective silencing, and the 2# siRNA had a higher silencing efficiency (figure 1f).

**Figure 2.**
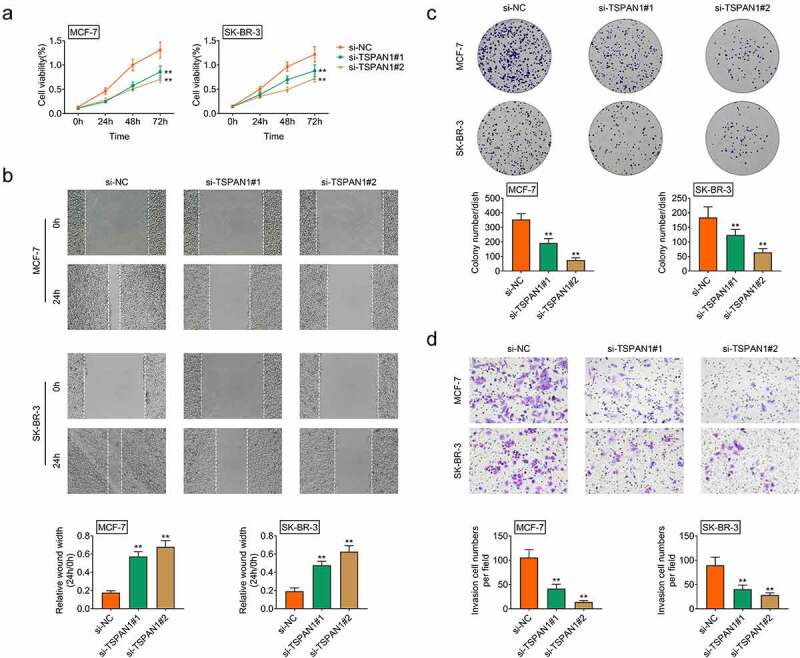
**TSPAN1 contributes to the growth and motility of breast cancer cells**. (a). CCK-8 assays showed the growth capacity of MCF-7 and SK-BR-3 cells upon the transfection of control or TSPAN1 siRNAs. (b). Wound closure assays showed the migration capacity of MCF-7 and SK-BR-3 cells upon the transfection of control or TSPAN1 siRNAs. The quantification of panel B, and the wound width was measured. (c). Colony formation assays confirmed the effects of TSPAN1 on the proliferation of MCF-7 and SK-BR-3 cells. (d). Transwell assays showed the invasion capacity of MCF-7 and SK-BR-3 cells upon the transfection of control or TSPAN1 siRNAs. Data are shown as mean ± SEM, *p < 0.05, **p < 0.01

Subsequently, we conducted CCK-8 assays to detect the effects of TSPAN1 on breast cancer growth. We noticed that TSPAN1 knockdown suppressed the growth of MCF-7 and SK-BR-3 cells (Figure 2a). In addition, we found that silencing of TSPAN1 suppressed the migration of cells, with the increased wound width (Figure 2b). Colony formation assays results showed that downregulation of TSPAN1 decreased the proliferation of MCF-7 and SK-BR-3 cells (Figure 2c). Similarly, we found TSPAN1 silencing restrained the invasion of both MCF-7 and SK-BR-3 cells, with the decreased invasion cell numbers (Figure 2d). Collectively, we thought TSPAN1 contributed to the growth and motility of breast cancer cells.

## Knockdown of TSPAN1 restrained the EMT process and PI3K/AKT pathway in breast cancer cells

**Figure 3.**
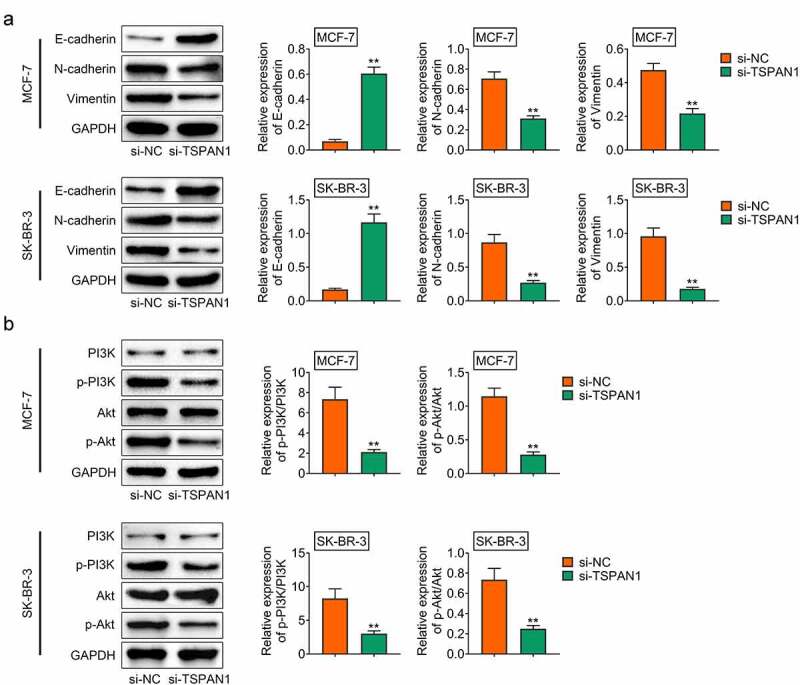
**Knockdown of TSPAN1 restrained the EMT process and PI3K/AKT pathway in breast cancer cells**. (a). Immunoblot assays showed the protein levels of E-cadherin, N-cadherin, Vimentin in MCF-7 and SK-BR-3 cells upon the transfection of control or TSPAN1 siRNAs. (b). Immunoblot assays showed the protein levels of PI3K and AKT, and their phosphorylation levels in MCF-7 and SK-BR-3 cells upon the transfection of control or TSPAN1 siRNAs. Data are shown as mean ± SEM, **p < 0.01

Since previous study indicated the effects of TSPAN1 on EMT process, we then performed Immunoblot assays to detect the effect of TSPAN1 on the EMT of breast cancer cells [18]. We detected the expression of E-cadherin and N-cadherin upon the transfection of TSPAN1 siRNA in breast cancer cells. Through Immunoblot, we noticed the increase in E-cadherin and decrease in N-cadherin after TSPAN1 silencing in these cells (Figure 3a). In addition, another EMT marker, Vimentin, was decreased after TSPAN1 silencing in MCF-7 and SK-BR-3 cells (Figure 3a). These results confirmed the effects of TSPAN1 on the EMT of breast cancer cells.

Subsequently, we detected the effects of TSPAN1 on the PI3K/AKT pathway in breast cancer cells. Through Immunoblot, we found that downregulation of TSPAN1 inhibited the phosphorylation levels of PI3K and AKT, suggesting that TSPAN1 mediated the PI3K/AKT pathway in breast cancer cells (Figure 3b).

## TSPAN1 promotes tumor growth of breast cancer in mice

**Figure 4.**
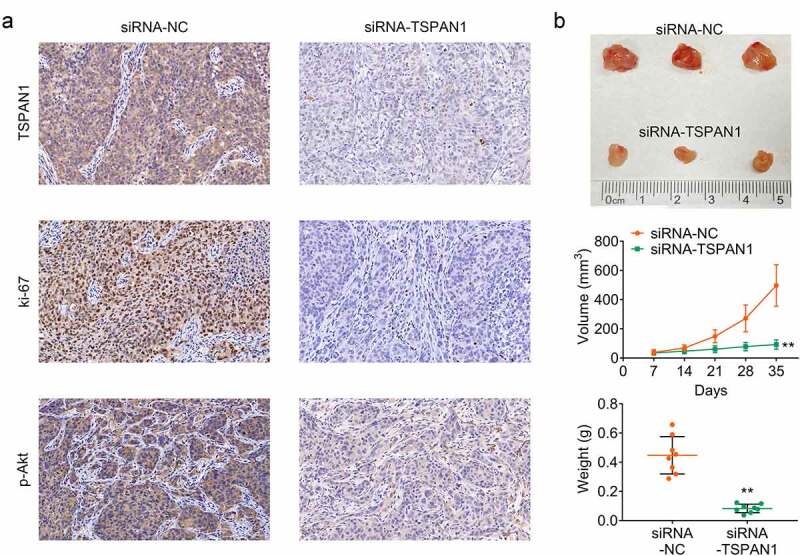
**TSPAN1 promotes tumor growth of breast cancer in mice**. (a) IHC assays showed the decreased levels of TSPAN1, Ki67, and p-AKT in control or TSPAN1 depletion tumor tissues. (b). Xenograft assay showed the tumor growth difference between control or TSPAN1 depletion mice. Data are shown as mean ± SEM, **p < 0.01

We further examined the role of TSPAN1 in breast cancer by xenograft study in mice. CAL27 cells transfected with TSPAN1 siRNA or control siRNA were subcutaneously inoculated into the upper back of nude mice and mice tail vein. Tumor volumes were calculated every 7 days to generate the growth curve. After 35 days, tumors were harvested, and mice were sacrificed. The results showed that tumor growth in the TSPAN1 siRNA group was slower than that of the control (Figure 4b). By immunohistochemistry analysis, we detected the expression of TSPAN1, Ki67, and the phosphorylation of AKT in tumor tissues, and found the decrease in TSPAN1 and Ki67 expression and the phosphorylation levels of AKT in TSPAN1-depleted tumor tissues (Figure 4a). Taken together, we demonstrated that silencing of TSPAN1 suppressed the growth of tumors via PI3K/ AKT pathway *in vivo*.

## Discussion

Breast cancer is a common malignant tumor that endangers women’s health. Since the end of 1970s, its incidence ranks the first place of female tumors, and it increases by 2% per year [19]. Advanced breast cancer is prone to distal metastasis, which greatly increases the difficulty of treatment [20]. Conventional surgical resection and chemoradiotherapy have little effect on breast cancer with high metastasis [21]. In order to further improve treatment outcomes of patients, new and more effective treatments need to be developed. For breast cancer, targeted therapy is very promising, but new therapeutic targets need to be explored and identified, and the pathogenesis needs to be further revealed [9]. In this study, we identified a member of the TSPAN superfamily, TSPAN1, which was abnormally high expressed in human breast cancer cells and tissues. We further investigated its role in the growth and motility of breast cancer cells *in vitro* and explored the mechanism. Since MCF-7 cell line was a metastatic cell line, we used it in the *in vivo* assays, and found the effects of TSPAN1 on tumor growth *in vivo*. Our data suggest that TSPAN1 could serve as a promising therapeutic target for breast cancer treatment.

Through CCK-8, wound closure, and transwell assays, we confirmed that TSPAN1 promoted the growth as well as motility of breast cancer cells. Through Immunoblot assays, we further revealed the effects of TSPAN1 on the EMT process and PI3K/AKT pathway in breast cancer cells. Also, TSPAN1 contributed to tumor growth of breast cancer cells in mice. These findings confirmed the key role of TSPAN1 in the progression of breast cancer. In fact, the effects of TSPAN1 on the progression of cancers have been widely revealed [18,22,23]. TSPAN1 could up-regulate matrix metalloproteinase 2 (MMP2) through PLCγ to promote migration and invasion of pancreatic cancer cells [12]. Another study indicated the effects of TSPAN1 on the autophagy of pancreatic cancer [24,25]. They showed that TSPAN1 promoted autophagy flux and mediated cooperation between WNT-CTNNB1 signaling and autophagy in pancreatic cancer [25]. In addition, TSPAN1 was under control of androgens and its upregulation increased the motility of prostate cancer cells [26]. Its depletion also suppressed the growth and infiltration of skin squamous carcinoma cells [27]. These studies provided the evidence that TSPAN1 could serve as a potential target for cancer treatment.

In this study, we also noticed the effects of TSPAN1 on the EMT process and PI3K/AKT pathway of breast cancer cells [24]. In fact, its effects on EMT and PI3K/AKT pathway have been previously revealed [18]. TSPAN1 promoted EMT and metastasis of cholangiocarcinoma by activating PI3K/AKT pathway, and we also revealed a similar mechanism in breast cancer [18]. EMT causes tumor epithelial cells to lose their polarity, converts adherent phenotypes into mesenchymal forms, and increases the ability of cancer cells to invade and migrate [28]. EMT also plays a key role in the metastasis of malignant tumor cells [29]. The EMT process was affected by the E-cadherin and N-cadherin, and the expression alterations of E-cadherin and N-cadherin could further affect EMT process and tumor development. PI3K/AKT pathway affects multiple types of cellular processes, such as growth, migration, EMT, as well as autophagy, and this pathway also affects the progression of multiple types of cancers, including breast cancer [30,31]. In the future study, we should confirm the effects of AKT inhibitors on TSPAN1-mediated breast cancer progression. Several proteins promote breast cancer progression through this pathway [31]. These studies, together with our findings, confirmed this pathway could serve as a promising target for breast cancer cells.

## Conclusion

In conclusion, we found the high TSPAN1 expression in breast cancer tumor samples and cell lines, and it was confirmed by bioinformation analysis. TSPAN1 knockdown suppressed the growth, and motility of breast cancer cells. We further found that TSPAN1 contributed to the EMT and mediated the PI3K/Akt pathway in breast cancer cells. In addition, TSPAN1 silencing suppressed tumor growth of breast cancer in mice. We thought TSPAN1 could serve as a potential target for breast cancer therapy.

## Highlight

TSPAN1 was high expression in human breast cancer.

TSPAN1 contributes to the growth and motility of breast cancer cells.

Knockdown of TSPAN1 restrained the EMT process and PI3K/AKT pathway in breast cancer cells.

TSPAN1 promotes tumor growth of breast cancer in mice.
